# A systematic review and meta-analysis for the association of the insulin-like growth factor1 pathway genetic polymorphisms with colorectal cancer susceptibility

**DOI:** 10.3389/fonc.2023.1168942

**Published:** 2023-05-22

**Authors:** Makan Cheraghpour, Masomeh Askari, Sascha Tierling, Sajad Shojaee, Amir Sadeghi, Pardis Ketabi Moghadam, Maryam Khazdouz, Hamid Asadzadeh Aghdaei, Moein Piroozkhah, Ehsan Nazemalhosseini-Mojarad, Nayeralsadat Fatemi

**Affiliations:** ^1^ Basic and Molecular Epidemiology of Gastrointestinal Disorders Research Center, Research Institute for Gastroenterology and Liver Diseases, Shahid Beheshti University of Medical Sciences, Tehran, Iran; ^2^ Department of Genetics/Epigenetics, Faculty NT, Saarland University, Saarbrücken, Germany; ^3^ Gastroenterology and Liver Diseases Research Center, Research Institute for Gastroenterology and Liver Diseases, Shahid Beheshti University of Medical Sciences, Tehran, Iran; ^4^ Growth and Development Research Center, Tehran University of Medical Sciences, Tehran, Iran

**Keywords:** insulin-like growth factor family, colorectal cancer, polymorphism, meta-analysis, insulin receptor substrate

## Abstract

**Background:**

The receptors, ligands, and associated proteins of the insulin-like growth factor (IGF) family are involved in cancer development. The *IGF1* receptor and its accompanying signaling cascade are a crucial growth-regulatory mechanism that plays an important role in colorectal cancer (CRC) proliferation and differentiation. *IRS1* (Insulin receptor substrate-1), a major substrate for the *IGF1R*, is involved in cell growth and promotes tumorigenesis. There are shreds of evidence from prior research suggesting that *IGF* system polymorphisms may influence susceptibility to CRC. However, the findings in this area were contradictory. Accordingly, we carried out a systematic literature search to identify all case-control, cross-sectional, and cohort studies on the association between various polymorphisms across four *IGF1* pathway genes (*IGF1*, *IGF1R*, *IRS1*, and *IRS2*) and the risk of CRC.

**Methods:**

We performed a comprehensive search strategy in PubMed, Scopus, and Web of Science databases for articles available until Aug 30, 2022. A total of 26 eligible studies with *IGF1*/*IGF1R*, *IRS1* and *IRS2* polymorphisms; met the inclusion criteria. All case-control studies for *IGF1* rs6214C>T, *IRS1* rs1801278G>A, and *IRS2* rs1805097G>A comprising 22,084 cases and 29,212 controls were included in the current meta-analysis. The pooled odds ratios (ORs) with 95% confidence intervals (CIs) were used to evaluate relationships between the polymorphisms and CRC susceptibility. All statistical analyses were performed using STATA software version 14.0.

**Results:**

The meta-analysis of available data for rs6214C>T, rs1801278G>A, and rs1805097G>A showed a significant association between these polymorphisms and an increased CRC risk in some of the comparisons studied (rs6214C>T, pooled OR for CC = 0.43, 95% CI 0.21- 0.87, P = 0.019; rs1801278G>A, OR for GA = 0.74, 95% CI 0.58-0.94, P = 0.016; rs1805097G>A, OR for GA = 0.83, 95% CI 0.71-0.96, P = 0.013). Nevertheless, the meta-analysis did not include other genetic variations in *IGF1, IGF1R, IRS1*, and *IRS2* due to heterogeneity and limited sample size.

**Conclusions:**

This systematic review and meta-analysis provide evidence that genetic variants in *IGF1* rs6214C>T, *IRS1* rs1801278G>A, and *IRS2* rs1805097G>A are associated with an increased risk of CRC. These findings may contribute to a better understanding of the complex genetic mechanisms involved in CRC development and could inform future research on prevention and treatment strategies for this disease.

## Introduction

The Insulin Growth Factor (IGF) protein family plays a key role in cell proliferation, apoptosis, and cell transformation through regulatory proteins synthesis (1). The Insulin Growth Factor-1 (IGF)-/Insulin-like Growth Factor-Receptor1 (IGF1R) pathway plays critical roles in the regulation of tumor cell metabolism, proliferation, survival, and angiogenesis (2, 3). IGF signaling pathway is activated when cell surface receptors like IGF1R bind to Insulin-like growth factors 1 and 2 (IGF1,2), and stimulate the phosphatidylinositol-3 kinase (PI3k)/Akt signaling pathway (4, 5). Any genetic alteration in IGF/IGF1R pathway members may result in insulin sensitivity (6).

This signaling pathway as a critical determinant has been linked to the development of colorectal cancer (CRC) (7, 8) and much evidence displayed hyperinsulinemia as a determinant of CRC risk, especially in those with younger onset (9–12). Consequently, evidence implicated the IGF1R and its ligands, IGF1, and IGF2, in tumor development and progression including CRC (13, 14).

Insulin receptor substrate (IRS) proteins including IRS1 and IRS2 are the major cytoplasmic molecules regulating the downstream signaling of IGF/IGF1R (15). They can interact with IGF receptors, leptin, vascular endothelial growth factors, growth hormone, prolactin, integrin, cytokine, and interferon receptors. These interactions display the critical role of IRS proteins in cancer development (16). In addition, these proteins activate and regulate intracellular signaling cascades including phosphatidylinositol 3-kinase/Akt (PI3K/Akt) and extracellular signal-regulated kinase (ERK) pathways that are involved in metabolism and protein synthesis, cell proliferation, and key regulators of CRC development and progression (17, 18).

Genetic variation in the insulin‐like growth factor (IGF) pathway would prove a role in IGF-related factors in colorectal tumorigenesis. Indeed, several studies revealed a significant association with CRC risk for genetic variants in genes encoding IGF-related factors (19–24). However, most single SNPs confer a small increase in the risk and the gene-gene and gene-environment interactions (25) and functional genetic compensation between genes may exist (26).

As mentioned above, the IGF pathway has been shown to play a critical role in the development and progression of CRC, and genetic variations in the pathway may contribute to CRC risk. However, there is limited integration of IGF pathway-linked genetic data in previous studies. This review and meta-analysis aim to address this gap in knowledge by systematically analyzing the available evidence on the associations between four IGF1 pathway gene (*IGF1*, *IGF1R*, *IRS1*, and *IRS2*) polymorphisms and CRC risk. The novelty of this study lies in the comprehensive analysis of multiple genetic variants across four genes in the IGF1 pathway, which may provide insights into the complex genetic mechanisms underlying CRC development and inform future research on the prevention and treatment of CRC.

## Materials and methods

### Literature search strategy

This systematic review and meta-analysis were performed based on Preferred Reporting Items for Systematic Reviews and Meta-Analyses (PRISMA) Statement (27). Searches were conducted in PubMed, Scopus, and Web of Science databases until Aug 30, 2022, using keywords shown in **Supplementary Text**. In the search, only English language and human studies were considered. Two reviewers independently searched the literature, screened titles, abstracts, and full texts, and consulted the third author whenever disagreements occurred.

### Inclusion and exclusion criteria

Inclusion criteria were: 1) Case-control, cross-sectional, and cohort studies in CRC populations; 2) Quantitative analysis of interplay between Genetic variants and CRC risk were reported; 3) Full text in the English language was available. Randomized controlled trials (RCT), reviews, letters, comments, editorials, case reports, conference abstracts, and personal communications, and studies focused solely on hereditary nonpolyposis colorectal cancer (HNPCC) were excluded.

### Data collection and assessment of the methodological quality

According to the inclusion criteria, two reviewers extracted the following data from the included studies: the name of the first author, year of publication, participant’s race and ethnicity, type of study, number of cases and control, family history of cancer, body mass index, age, *KRAS* status, gene and variation genotype, clinical outcome, type of drug, and CRC stages. The quality of each study was assessed using Critical Appraisal Skills Programme (CASP) checklist by three reviewers (**Supplementary Table 1**). As part of the screening process and quality assessment, conflicts were resolved with the fourth author through discussions or consultation.

### Statistical analysis

Meta-analyses were performed on the extracted data for dichotomous outcome variables of colorectal cancer. We calculated a pooled odds ratio (OR) and 95% confidence intervals (CI) for several genetic variations including *IGF1* (969(CA), rs6214C>T, rs35767C>T), *IRS1* (rs1801278G>A), and *IRS2* (rs1805097G>A). We applied fixed or random effects of meta-analyses due to heterogeneity with the inverse variance (IV) weighting in overall analysis by Forest plots. If heterogeneity was rejected, a random model was used to calculate pooled estimates. Heterogeneity of variances was assessed using Cochran’s Q test and I^2^ measure and was plotted with a radial diagram (28). We also assessed publication bias by a funnel plot and calculating Egger’s test (29). All statistical analysis was conducted using STATA, version 14.0 (Stata Corp, College Station, TX).

## Results

### Search result, study characteristics, and quality assessment

**Figure 1:**
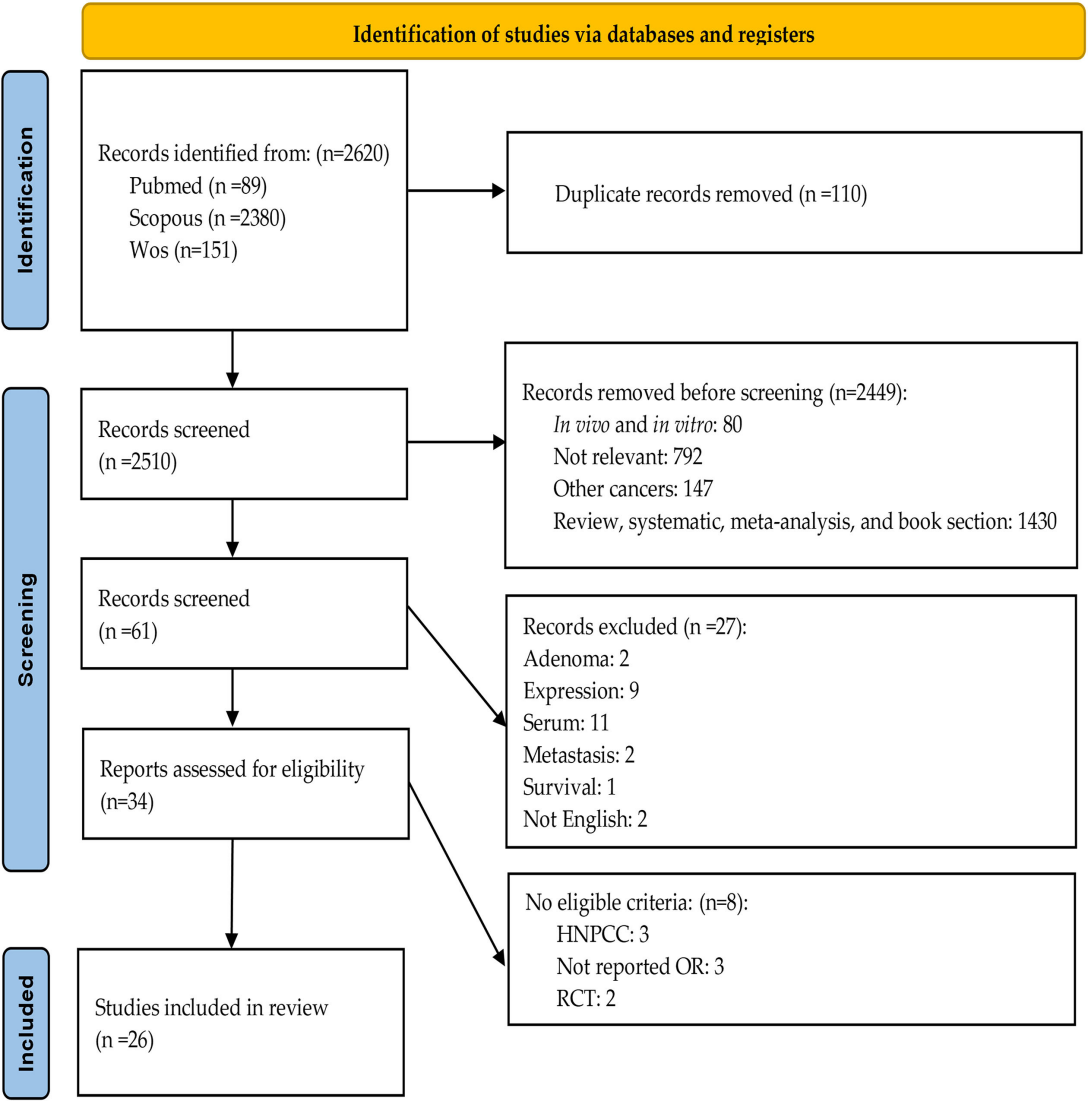
Flow chart for studies included in this systematic review.

A total of 2620 articles were retrieved through electronic and manual searches. Title and abstract reviews led to the removal of duplicates (n=110) and the exclusion of articles that were not in English or did not meet our inclusion criteria (n=2510) (**Figure 1**). Baseline characteristics of the included manuscripts are presented in **Table 1**. A quality appraisal determined that all inquiries were high-quality studies.

**Table 1:** Characteristics of the included studies.

No.	Authors and year of publication	Type of study	Ethnicity	Country	Case	Control	Gene	SNP/microsatellite IDs
**1**	Wong, H L et al. (30)	Case-control	Chinese	Southern China	290 (122 F, 168 M)	873 (492 F, 381 M)	IGF1	-969(CA) repeat
**2**	Jen-Kou Lin, et al. (31)	cohort	_	Taiwan	950	_	IGF1	rs12579108C>A
**3**	Martha L Slattery et al. (32)	Case-control	Non-Hispanic White	USA	Colon: 1346 (590 F, 756 M) Rectal: 952 (393F, 559 M)	Colon: 1544 (699 F, 845 M) Rectal: 1205 (532 F, 673 M)	IGF1, IRS1, IRS2	-969(CA) repeat, rs1801278G>A, rs1805097G>A
**4**	Libby M Morimoto et al. (33)	Case-control	Non-Caucasian	USA	782 (447F, 335 M)	503 (350F, 153 M)	IGF1	-969(CA) repeat
**5**	Wade S Samowitz et al. (34)	Case-control	African American, white, or Hispanic	USA	1788 (987 M, 801 F)	1981 (1060 M, 921 F)	IGF1, IRS1, IRS2	-969(CA) repeat, rs1801278G>A, rs1805097G>A
**6**	Sonali Pechlivanis et al. (35)	Case-control	Czechs	Czech Republic	712 (404 M, 308 F)	748 (434 M, 314F)	IRS1, IRS2	rs1801278G>A, rs1805097G>A
**7**	Sonali Pechlivanis et al. (36)	Case-control	German	Germany	661	607	IGF1	-969(CA) repeat, rs7136446C>A, rs35767C>T
**8**	Armin Gerger et al. (37)	cohort	_	USA	115	_	IGF1	rs6220A>G
**9**	Ayman Yosry et al. (38)	Case-control	Egyptian	Egypt	66 (45 M, 21 F)	30 (16 M, 14 F)	IGF1	rs35767C>T, rs6214C>T, rs6220A>G
**10**	Elisabeth Feik et al. (39)	Case-control	Caucasian	Austria	178 (106 M, 72F)	1795 (838M, 957F)	IGF1	rs35767C>T, rs6214C>T, rs6220A>G
**11**	Shahad W. Kattan et al. (40)	Case-control	_	Saudi Arabia	80 (47M, 33F)	80 (43M, 37F)	IGF1	rs6214C>T
**12**	Touraj Mahmoudi et al. (41)	Case-control	Iran		261 (146M, 115F)	339 (164M, 175F)	IGF1, IRS1, IRS2	rs5742612T>C, rs1801278G>A, rs1805097G>A
**13**	Yoon Young Cho et al. (42)	cohort	Korea		402 (217 M, 185 F)	_	IGF1	rs2288378T>C, rs6220A>G, rs5742612T>C, rs5742714C>G, rs12579108C>A
**14**	Khatoon Karimi et al. (43)	Case-control	Iranian	Iran	167 (91 M, 76 F)	277 (128 M, 149 F)	IGF1, IRS2	rs6214C>T, rs2289046A>G
**15**	Touraj Mahmoudi et al. (44)	Case-control	Iranian	Iran	312 (174M, 138 F)	438 (221M, 217F)	IRS1	rs1801278G>A
**16**	Emel Hulya Yukseloglu et al. (45)	Case-control	Turkish	Turkey	161 (94 M, 67 F)	197 (95M, 102F)	IRS2	rs1805097G>A
**17**	Nicholas J Ollberding et al. (46)	Case-control	Multiethnic	USA	1954 (1,082M, 872F)	2587 (1503M, 1084F)	IGF1	rs35767C>T
**18**	H-L Wong et al. (47)	Case-control	Chinese Singaporeans	USA	298 (169M, 129 F)	1142 (489M, 653F)	IGF1	rs12579108C>A
**19**	Noyko S Stanilov, et al. (48)	Case-control	Caucasian	Bulgaria	110 (67M, 43F)	143	IGF-1R	rs2229765G>A
**20**	Jennie Ong et al. (49)	Case-control	Caucasian	Netherlands	544 (325M, 219F)	544 (325M, 219F)	IGF1	rs6214C>T
**21**	Temitope O Keku et al. (19)	Case-control	Americans	USA	528	836	IGF1	-969(CA) repeat
**22**	Colinda C J M Simons et al. (21)	cohort	_	Netherlands	3440	_	IGF1, IRS1, IRS2	-969(CA) repeat, rs1520220C>G rs5742678C>G rs10735380G>A rs5742694G>T
**23**	Xianyang Li et al. (50)	Case-control	Chinese	China	208	312	IGF1	rs35767C>T
**24**	Yang Li et al. (23)	Case-control	Chinese	China	367 (219M, 148F)	367 (229M, 138F)	IGF1	rs35767C>T
**25**	X L Chao et al. (51)	Case-control	Chinese	China	367 (219M, 148F)	367 (229M, 138F)	IGF1	-969(CA) repeat
**26**	Thomas Winder et al. (52)	Cohort	_	USA	130 (64M, 66F)	_	IGF1, IGF1R	rs6214C>T, rs6220A>G, rs2946834G>A, rs7136446C>T, rs2272037T>C, rs2016347G>T, rs2229765G>A

(-), Not Reported.

There were 23 studies that analyzed the association between *IGF1* and *IGF1R* polymorphisms and CRC, 20 of which were case-control studies and three were cohort studies. A total of eight studies reported associations between *IRS1* and *IRS2* polymorphisms and CRC, but only one of them was a cohort study.

### IGF1, IGF1R, IRS1, and IRS2 polymorphisms and CRC risk

#### IGF1

Three *IGF1* polymorphisms (-969(CA) repeat, rs35767C>T, and rs6214C>T) have been most intensely investigated (**Table 2**). The CA repeat polymorphism, which is located 969 bp upstream from the transcription start site, has been investigated in six studies. Wong et al. found no association between risks of CRC and *IGF1* genotypes [-969(CA)19, -969(CA)18] in the 290 case and 873 control from Chinese population. However, they found that CA(21) was associated with CRC risk (P=0.02) (30).

**Table 2:** Stratified analyses of the *IGF1* polymorphism on CRC risk and circulating level of IGF1.

SNP	Location	Genotype	Association results		Tumor site	Function	Ref
			OR (95% CI)	P-Value			
-969(CA) repeat	969 bp upstream from the transcription start site	Others	1.00	_	CRC	Association between risks of CRC and (CA)21	(30)
		(CA) 19/others	1.33 (0.98-1.80)				
		(CA)19/(CA)19	OR 1.03 (0.64-1.63)				
		(CA)21/others	OR 0.90 (0.67,1.23)	**0.02**	CRC		
		(CA)21/(CA)21	OR 0.46 (0.26,0.81)				
		Others	1.2 (0.9-1.5)	NR	Colon	No association between risks of CRC and *IGF1* genotypes	(32)
			0.9 (0.7-1.2)		Rectal		
		(CA) 19/others	1.2 (1.0-1.4)		Colon		
			1.0 (0.8-1.2)		Rectal		
		(CA)19/(CA)19	1.00		Colon		
			1.00		Rectal		
		Others	1.5 (1.1, 2.0)	NR	CRC	No association between risks of CRC and *IGF1* genotypes	(34)
		(CA) 19/others	1.1 (0.9, 1.4)				
		(CA)19/(CA)19	1.0				
		Others	1.3 (1.0-1.6)	**< 0.05**	CRC	Association between CA repeat except homozygous CA(19) and increased risks of CRC	(33)
		(CA)19/(CA)19	1.0				
		Others	0.84 (0.59–1.20)	NR	CRC	No association between CA(19) genotype and CRC risk	(35)
		(CA) 19/others	1.18 (0.91–1.51)				
		(CA)19/(CA)19	1.0				
		Others (AA)	1.0	NR	CRC	Association between CA(19) homozygous genotype and CRC risk in White race, No significant association between (CA)19 repeat and circulating level of IGF1	(19)
		Others (W)	1.0				
	(CA) 19/others (AA)	0.87 (0.59–1.27)					
		(CA) 19/others (W)	1.09 (0.73–1.63)				
		(CA)19/(CA)19 (AA)	0.73 (0.50–1.51)				
		(CA)19/(CA)19 (W)	1.77 (1.15–2.73)				
		Others (M)	1.05 (1.03, 1.07) ^*^	0.98	CRC	Association of CA (19) allele with lower CRC risks in female	(21)
		Others (F)	0.54 (0.42, 0.70) ^*^	**< 0.001**			
		(CA) 19/others (M)	1.02 (0.81, 1.10) ^*^	0.98			
		(CA) 19/others (F)	0.84 (0.67, 1.06) ^*^	**< 0.001**			
		(CA)19/(CA)19 (M)	1.0	0.98			
		(CA)19/(CA)19 (F)	1.0	**< 0.001**			
		Others (M)	0.96 (0.74, 1.24) ^*^	0.67	Colon		
		Others (F)	0.50 (0.38, 0.65) ^*^	**< 0.001**			
		(CA) 19/others (M)	0.92 (0.74, 1.15) ^*^	0.67			
		(CA) 19/others (F)	0.79 (0.62, 1.02) ^*^	**< 0.001**			
		(CA)19/(CA)19 (M)	1.0	0.67			
		(CA)19/(CA)19 (F)	1.0	**< 0.001**			
		Others (M)	1.03 (0.73, 1.46) ^*^	0.97	Rectal		
		Others (F)	0.84 (0.52, 1.38) ^*^	0.46			
		(CA) 19/others (M)	0.87 (0.64, 1.18) ^*^	0.97			
		(CA) 19/others (F)	1.14 (0.72, 1.81) ^*^	0.46			
		(CA)19/(CA)19 (M)	1.0	0.97			
		(CA)19/(CA)19 (F)	1.0	0.46			
		Others	0.76 (0.50-1.17)	0.209	CRC	Association of CA (19) allele with lower CRC risks in male, No significant association between (CA)19 repeat and circulating level of IGF1.	(51)
		(CA) 19/others	0.71 (0.46-1.10)	0.126			
		(CA)19/(CA)19	1.0	NR			
rs35767C>T	5’UTR	CC	1.0	NR	CRC	No significant association with CRC risk	(35)
		CT	1.03 (0.79–1.33)				
		TT	1.18 (0.59–2.38)				
		CC	OR:1.76 (1.03 -3.01)	NR	CRC	No significant association with CRC risk	(38)
		CT or TT	1.83 (0.73 - 4.59)				
		CC	1.0	NR	CRC	No significant association with CRC risk	(39)
		CT	1.26 (0.85–1.89)	0.25			
		TT	0.58 (0.14–2.45)	0.46			
		CC	1.0	NR	CRC	No significant association with CRC risk, Associations with circulating IGF-I levels	(46)
		CT	0.94 (0.83–1.07)				
		TT	0.81 (0.66–1.01)				
		CC	1.0	NR	CRC	Association of AA genotype with CRC risk	(50)
		CT	0.96 (0.65–1.42)	0.92			
		TT	2.26 (1.35–3.80)	**0.003**			
		CC	1.0	NR	CRC	Association of CT genotype with CRC risk, Association of CC, CT, and TT genotype with higher IGF-1 levels in the CRC group	(23)
		CT	1.399 (1.029-1.901)	**0.032**			
		TT	1.213 (0.734-2.005)	0.451			
rs6214C>T	3’UTR	CC	1.0	NR	CRC	Association of TT genotype with CRC risk	(39)
		CT	1.27 (0.83–1.95)	0.28			
		TT	1.79 (1.04–3.08)	**0.04**			
		CC	1.0	NR	CRC	No significant association with CRC risk	(43)
		CT	0.846(0.542–1.321)	0.463			
		TT	0.749 (0.395–1.423)	0.378			
		CC	1.0	NR	CRC	No significant association with CRC risk	(49)
		CT	1.04 (0.80–1.36)	0.750			
		TT	1.21 (0.84–1.74)	0.311			
		CC	1.41 (0.88 - 2.27)	NR	CRC	Association of CT or TT genotype with CRC risk	(38)
		CT or TT	17.68 (2.27 - 137.99)				
		CC	1.0	NR	CRC	Association of CT and TT genotype with CRC risk and highest levels of serum IGF-1 levels	(40)
		CT	8.333(3.40–20.48)	**< 0.001**			
		TT	10.417 (4.42–24.52)	**< 0.001**			
rs6220A>G	3’UTR	AA	1.0	NR	CRC	No significant association with CRC risk	(39)
		AG	1.22 (0.83–1.80)	0.32			
		GG	0.93 (0.43–2.00)	0.64			
		AA	3.50 (1.60 - 7.68)	NR	CRC	No significant association with CRC risk	(38)
		AG or GG	0.49 (0.19 - 1.27)				
rs12579108C>A	Promoter	CC	1.0	NR	Colon	Association of AA and CA genotype with decreased CRC risk	(47)
		CA	0.51 (0.36 to 0.73)				
		AA	0.59 (0.33 to 1.04)				
		CC	1.0	NR	Rectal		
		CA	0.65 (0.43 to 0.98)				
		AA	0.83 (0.45 to 1.53)				
rs5742612T>C	Promoter	TT	1.0	NR	Colon	Association of CT and CC genotype with decreased CRC risk	(30)
		CT	0.59 (0.40-0.86)				
		CC	0.54 (0.29-0.99)				
		TT	1.0	NR	Rectal		
		CT	0.91 (0.59-1.37)				
		CC	1.08 (0.60-2.00)				
		TT	1.0	NR	CRC	No significant association with CRC risk	(41)
		CT	0.92 (0.39–2.17)				
		CC	0.62 (0.03–11.10)				
rs7136446C>A	Intron	AA	1.0	NR	CRC	No significant association with CRC risk	(35)
		AG	1.04 (0.80–1.34)				
		GG	1.06 (0.75–1.48)				
rs1520220C>G	Intron	CC (M)	1.0	**0.04**	CRC	Association with CRC risks in male	(21)
		CC (F)	1.0	0.98			
		CG (M)	1.15 (0.97, 1.35) ^*^	**0.04**			
		CG (F)	1.00 (0.83, 1.19) ^*^	0.98			
		GG (M)	1.37 (0.92, 2.05) ^*^	**0.04**			
		GG (F)	1.03 (0.65, 1.63) ^*^	0.98			
rs5742678C>G	Intron	CC (M)	1.0	0.05	CRC	No significant association with CRC risk	(21)
		CC (F)	1.0	0.97			
		CG (M)	1.14 (0.98, 1.34) ^*^	0.05			
		CG (F)	1.01 (0.85, 1.20) ^*^	0.97			
		GG (M)	1.25 (0.92, 1.69) ^*^	0.05			
		GG (F)	0.98 (0.69, 1.37) ^*^	0.97			
rs10735380G>A	Intron	AA (M)	1.0	0.09	CRC	No significant association with CRC risk	(21)
		AA (F)	1.0	0.26			
		AG (M)	1.10 (0.94, 1.28) ^*^	0.09			
		AG (F)	1.14 (0.96, 1.35) ^*^	0.26			
		GG (M)	1.25 (0.93, 1.67) ^*^	0.09			
		GG (F)	1.06 (0.76, 1.47) ^*^	0.26			
rs5742694G>T	Intron	TT (M)	1.0	**0.02**		Association with CRC risks in male	(21)
		TT (F)	1.0	0.95			
		GT (M)	1.13 (0.97, 1.33) ^*^	**0.02**			
		GT (F)	1.03 (0.86, 1.22) ^*^	0.95			
		GG (M)	1.38 (1.01, 1.88) ^*^	**0.02**			
		GG (F)	0.96 (0.67, 1.37) ^*^	0.95			

CRC, Colorectal cancer; M, Male; F, Female; AA, African American; W, White; NR, Not Reported.

*Hazard Ratio (HR); (-), Not Reported.The bold values show P-value lower than 0.05 that is statistically significant.

It was demonstrated that every genotype of CA repeat, except homozygous CA(19), had increased risks of CRC in 782 American patients (p<0.05) (33). Also, these genotypes modulated the association of BMI, physical activity, and consumption of hormones in postmenopausal women with CRC risk in women with the 19/19 genotype (33). In a study on 5047 subjects, 1346 with colon cancer, 952 with rectal cancer, and 2217 healthy controls, the correlation of CA(19) repeat with risk of CRC was analyzed. No significant association in either colon or rectal cases was found (32). Samowitz et al. conducted a multicenter study to assess the impact of CA(19) repeats on the risk of CRC. According to these results, there was no significant association between CA(19) repeats and 1788 cases of CRC (34). In line with this evidence, Pechlivanis et al. also observed no association between CA(19) genotype and CRC risk in the 661 case and 667 control from German population (35). A case-control study including 528 case and 836 control by Keku et al. examined the distribution of CA(19) repeat genotypes among two races. It was found that homozygous CA(19) genotype and colon cancer risk was associated in whites (p-value not reported), but not in African Americans (19). In cohort studies including 3440 CRC cases, it was proven that females carrying less than 38 repeats of the CA (19) allele had lower CRC risks at all subsites (p<0.001), except the rectum compared to those carrying more than 38 repeats (21). Conflicting results have been reported by Chao et al. who found CA(19) genotype with less than 38 repeats was associated with reduced CRC risk in 219 males (p-value not reported), but no interaction between CA(19) and CRC stage appeared to exist (51).

Six studies evaluated the correlation of rs35767C>T located in the 5’UTR of *IGF1* and CRC risk (**Table 2**). Four studies conducted on different populations, including German, Caucasians, Americans, and Egyptians, found no significant association of rs35767C>T with CRC risk (35, 38, 39, 46). However, two recent studies have reported the opposite results. Li et al.  found an increased risk of CRC for CT genotype carrier in 367 Chines cases (CT vs. CC, OR=1.399, 95% CI 1.029-1.901 P = 0.032), although no significant differences between genotype and stage were shown (23). A possible role for rs35767C>T in the risk of CRC has been suggested by Li et al. reported an association of TT genotype with CRC risk in 208 Chines cases (TT vs. CC: OR = 2.26, 95% CI = 1.35–3.80, P = 0.003). Furthermore, it was also found that in advanced tumor stages the incidence of TT or TC genotype is significantly higher (50).

For rs6214C>T that located in the 3’UTR of exon 4 in *IGF1*, five studies reported its relationship with CRC risk in different populations. Feik et al. found an increased risk of CRC for the carriers of TT genotype (OR = 1.79, 95% CI 1.04–1.90) in a cohort of 178 Caucasian cases (39), a finding confirmed in two other studies. Patients with TT or TC genotype had an increased risk of CRC in the Egyptian (OR 17.68, 95% CI; 2.27 - 137.99) (38) and Arab (p < 0.001) population (40). However, the association between rs6214C>T and CRC risk was not confirmed by two other studies (43, 49).

A study, on 178 patients found no significant association between rs6220A>G and risk of CRC (39). These results were confirmed by another case-control study conducted by Yosry et al. included a total of 66 patients to evaluate an association between rs6220A>G and risk of CRC. However, the distribution of the rs6220A>G genotype was not different between cases and controls (38).

As for other polymorphisms located in the promoter region of *IGF1*, some studies have investigated the role of rs5742612T>C and rs12579108C>A. In 290 Chines patients, CC and CT genotypes of rs5742612T>C were associated with a decreased risk of CRC (P-value not reported), and a protective effect of the C allele was observed among participants younger than 60 years (30). However, Mahmoudi et al. found no association of rs5742612T>C with risk of CRC in 261 Iranian patients (41). The rs12579108C>A has been investigated by one group, which found genotypes AA and CA considerably associated with reduced colorectal cancer risk (P-value not reported). The association was also significantly more substantial in the subgroup of patients with colon cancer compared to patients with rectal cancer (heterogeneity p<0.001) (47). 950 CRC patients enrolled in a cohort study and genotype distribution of rs12579108C>A were investigated between old and young patients. It was demonstrated that the frequency of the AA genotype of rs12579108C>A was 12.7%, which was significantly higher than young patients (31).A further SNP that was investigated only in one case-control study was rs7136446C>A. The results showed that rs7136446C>A was not associated with CRC risk in the German population (35). Four SNPs, including rs1520220C>G, rs5742678C>G, rs10735380G>A, and rs5742694G>T have been investigated in a large cohort study. Simons et al. found that rs5742694G>T (P=0.02) and rs1520220C>G (P=0.04) were associated with an increased risk of CRC in men (21).

#### Associations of IGF1 polymorphism with circulating IGF1 concentrations

The association between *IGF1* genotype and circulating level of IGF1 was assessed in five studies. A case–control study from a multiethnic Cohort showed that the rs35767C>T was associated with circulating IGF1 levels (P= 0.001) (46). A similar observation was made by Li et al. when they found higher IGF1 levels for three genotypes in the CRC group than for the control group (P<0.05) (23).

A study of 1364 subjects found that plasma concentrations of IGF1 were not significantly affected by the genotype of the CA(19) polymorphism (19). Supporting results have also been reported that CA(19) repeat polymorphism did not have any significant association with circulating IGF1 levels (51).

The results of a study involving 80 colorectal cancer patients and 80 matched controls revealed that TT and CT genotypes of rs6214C>T had the highest serum IGF1 levels (P= < 0.001) (40).

#### IGF1R

A study carried out by Stanilov et al. examined the relationship between the rs2229765G>A polymorphism in the *IGF1R* gene and the risk of CRC as well as the activity of the disease (**Table 3**). The genotypes AA or AG were more prevalent among 110 advanced CRC Caucasian cases as compared to controls (AA/AG vs. GG: OR= 3.06, P= 0.004) (48).

**Table 3:** Stratified analyses of the *IGF1R* polymorphisms on CRC risk.

SNP	Location	Genotype	Association results OR (95% CI) P-Value		Tumor site	Function	Ref
rs2229765G>A	Exon	AA	1.02 (0.37–2.81) 0.958	0.958	Early CRC	Association of AA or AG genotypes with advanced CRC	(48)
		AG	0.89 (0.43–1.84)	0.738			
		GG	1.0	NR			
		AA	3.20 (1.04–9.97)	**0.021**	Advanced CRC		
		AG	3.01 (1.25–7.44)	**0.007**			
		GG	1.0	NR			

The bold values show P-value lower than 0.05 that is statistically significant.

#### IRS1

The rs1801278G>A polymorphism (Gly972Arg) located in exon 1 of *IRS1* has been assessed in six different studies, two of which have found an association of this polymorphism with CRC risk (**Table 4**). One of the significant reported results was in the case-control study of Slattery et al., who demonstrated that having at least one minor allele (GA or AA) was associated with an increased risk of colon cancer (OR 1.4, 95% CI 1.1-1.9). Furthermore, individuals without a family history of colon cancer were found to have an increased risk of colon cancer if they carried the GA/AA genotype (32). In line with this evidence, the multicenter study of 1788 American cases and 1981 healthy control found a significantly increased risk of CRC in the GA/AA genotypes carrier (OR 1.3, 95% CI 1.0-1.5) (48). However, the results of four studies including, three case-control and one cohort study, did not support an association between rs1801278G>A and CRC risk (21, 32, 41, 44).

**Table 4:** Stratified analyses of the *IRS1* polymorphisms on CRC risk.

SNP	Location	Genotype	Association results		Tumor site	Function	Ref
			OR (95% CI)	P-Value			
rs1801278G>A	Exon	GG	1.0	NR	Colon	Association of GA or AA with an increased risk of colon cancer	(32)
			1.0		Rectal		
		GA/AA	1.4 (1.1-1.9)		Colon		
			1.1 (0.8-1.5)		Rectal		
		GG	1.0	0.568	CRC	No association with risk of CRC	(36)
		GA	0.85 (0.61–1.19)				
		AA	0.56 (0.06–5.44)				
		GG	1.0	NR	CRC	No association with risk of CRC	(41)
		GA	0.92 (0.51–1.64)	0.767			
		AA	1.09 (0.05–23.70)	0.955			
		GG (M)	1.0	0.58	CRC	No association with risk of CRC	(21)
		GG (F)	1.0	0.66			
		GA (M)	1.11 (0.90, 1.37) ^*^	0.58			
		GA (F)	1.03 (0.80, 1.31) ^*^	0.66			
		AA (M)	0.60 (0.20, 1.77) ^*^	0.58			
		AA (F)	2.33 (0.47, 11.65) ^*^	0.66			
		GG	1.0	NR	CRC	No association with risk of CRC	(44)
		GA	0.74 (0.43-1.27)	0.278			
		GA/AA	1.3(1.0, 1.5)	NR	CRC	Association of GA/AA with an increased risk of CRC	(34)

CRC: Colorectal cancer, Male: M; Female: F; Not Reported: NR.

*Hazard ratio (HR).

#### IRS2

Among *IRS2* polymorphisms, rs1805097G>A has been evaluated in six studies (**Table 5**). Three of them have found that rs1805097G>A was not associated with CRC in Caucasian, Turkish and Iranian populations (36, 41, 45). The only significant result reported was in the study conducted by Slattery et al. who found an association of GA genotype with risk of CRC in 1346 colon cancer patients (OR 0.8, 95% CI 0.6-0.9) (32). The other study of the American population indicated that rs1805097G>A was not associated with CRC (34). Furthermore, a cohort study was performed on a total of 3440 CRC patients from the Netherlands, confirming that not only rs1805097G>A but also rs2289046A>G, rs754204C>T, and rs4773082T>C were not associated with risk of CRC (21). Karimi et al. assessed the genotype distribution of rs2289046A>G within 167 Iranian CRC patients. Although the genetic association of rs2289046A>G with CRC risk was excluded, GG genotype was associated with reduced risk of CRC in subjects in the normal range weight (p=0.035, OR=0.259, 95%CI=0.074-0.907) (43).

**Table 5:** Stratified analyses of the IRS2 polymorphisms on CRC risk.

SNP	location	Genotype	Association results		Tumor site	Function	Ref
			OR (95% CI)	P-Value			
rs1805097G>A	Exon	GG	1.0	NR	Colon	An association of GA genotype with risk of CRC	(32)
			1.0		Rectal		
		GA	0.8 (0.6-0.9)		Colon		
			1.0 (0.9-1.3)		Rectal		
		AA	1.0 (0.7-1.3)		Colon		
			0.9 (0.7-1.2)		Rectal		
		GG	1.0	0.451	CRC	No significant association with risk of CRC	(36)
		GA	1.15 (0.89–1.48)				
		AA	0.97 (0.68–1.38)				
		GG	1.0	NR	CRC	No significant association with risk of CRC	(45)
		GA	0.76 (0.48–1.19)				
		AA	1.11 (0.58–2.11)				
		GG	1.0	NR	CRC	No significant association with risk of CRC	(41)
		GA	1.04 (0.71–1.51)	0.854			
		AA	0.97 (0.56–1.66)	0.898			
		GG (M)	1.15 (0.90, 1.47)^*^	0.20	CRC	No significant association with risk of CRC	(21)
		GG (F)	1.07 (0.82, 1.40) ^*^	0.51			
		GA (M)	1.07 (0.84, 1.36) ^*^	0.20			
		GA (F)	1.01 (0.77, 1.32) ^*^	0.51			
		AA (M)	1.0	0.20			
		AA (F)	1.0	0.51			
rs2289046A>G	3’UTR	AA	1.0	NR	CRC	No significant association with risk of CRC	(43)
		AG	1.177(0.777–1.782)	0.441			
		GG	0.678 (0.334–1.374)	0.281			
		AA (M)	1.20 (0.93,1.55)	0.15	CRC	No significant association with risk of CRC	(21)
		AA (F)	1.03 (0.78,1.34) ^*^	0.57			
		AG (M)	1.13 (0.88, 1.46) ^*^	0.15			
		AG (F)	0.94 (0.72, 1.23) ^*^	0.57			
		GG (M)	1.0	0.15			
		GG (F)	1.0	0.57			
rs754204C>T	Intron	CC (M)	1.0	0.14	CRC	No significant association with risk of CRC	(21)
		CC (F)	1.0	0.78			
		TC (M)	1.08 (0.91, 1.30) ^*^	0.14			
		TC (F)	0,98 (0.81, 1.20) ^*^	0.78			
		TT (M)	1.17 (0.95, 1.45) ^*^	0.14			
		TT (F)	1.04 (0.82, 1.31) ^*^	0.78			
rs4773082T>C	_	TT (M)	1.0	0.27	CRC	No significant association with risk of CRC	(21)
		TT (F)	1.0	0.81			
		CT (M)	1.11 (0.93, 1.33) ^*^	0.27			
		CT (F)	1.00 (0.82,1.23) ^*^	0.81			
		CC (M)	1.12 (0.89, 1.41) ^*^	0.27			
		CC (F)	1.03 (0.80, 1.33) ^*^	0.81			

CRC, Colorectal cancer; M, Male; F, Female; NR, Not Reported.

*Hazard Ratio (HR); (-), Not Reported.

#### Association of IGF1 and IGF1R polymorphisms with PFS and OS

A cohort study by Winder et al. investigated the association between polymorphisms of the *IGF1* and *IGF1R* genes with clinical outcome of 130 metastatic CRC (mCRC) patients treated with cetuximab monotherapy. A significant correlation was found between three *IGF1* polymorphisms, rs6214C>T (P=0.048), rs2946834G>A (p<0.001), and rs7136446C>A (P=0.034) with PFS (1.3 months, 95% CI, 1.3-1.5). In addition, PFS for mCRC with wt *KRAS* was independently predicted by two polymorphisms of *IGF1*, including rs2946834G>A (P=0.001) and rs713664 (P=0.022). As a result of the OS analysis, *IGF1* (rs7136446C>T), *IGF1R* (rs2272037T>C and rs2016347G>T) were associated with shorter OS in all patients (P=0.026, P=0.039, P=0.038 respectively), while in patients with wt *KRAS* only *IGF1R* (rs2016347G>T) significantly predicted shorter OS (P=0.004). Additionally, it was found that *IGF1* rs6214C>T, and rs2946834G>A, and *IGF1R* rs2016347G>T were negative predictors of cetuximab efficacy in mCRC patients (52). In another cohort study conducted by Cho et al., 440 Korean CRC patients were subjected to an analysis of the association of rs2288378T>C, rs6220A>G, rs5742612T>C, rs5742714C>G, and rs12579108C>A with OS and PFS. However, no correlation was observed between the distribution of genotypes of polymorphisms and OS and PFS in these patients (42). The role of *IGF1* and *IGFR1* polymorphism on OS and PFS was also studied in 132 patients treated with first-line bevacizumab (BV) and FOLFOX or XELOX. Gerger et al. provide evidence that patients carrying the AG or GG genotype of rs6220A>G showed a median OS of 32.4 months, while those carrying AA genotype had a median OS of 22 months (HR 0.51; 95%CI 0.32–0.83) (37).

#### Meta-analysis result

The results of the meta-analysis on the association of *IGF1* rs6214C>T, *IRS1* rs1801278G>A, and *IRS2* rs1805097G>A polymorphisms with CRC risk are shown in **Table 6**.

**Table 6:** Association between IGF1 pathway genes polymorphisms and CRC risk.

Gene	SNP	Studies (N)	Cases (N)	Controls (N)	Genotype	OR (95% CI)	P
IGF1	969(CA)	**7** (19, 30, 32–35, 51)	7,024	8,519	CA(19)/CA(19)	0.976 (0.85-1.11)	0.721
					CA(19)/non(19)	0.934 (0.72-1.19)	0.587
					non(19)/non(19)	1 (0.92-1.09)	0.864
	rs6214C>T	**5** (38–40, 43, 49)	1,035	2,726	CC	0.43 (0.21-0.87)	**0.019**
					CT	0.97 (0.64-1.47)	0.879
					TT	1.37 (0.83-2.26)	0.216
	rs35767C>T	**6** (35), 23, 38, 39, 46, 50)	3,434	5,698	CC	0.94 (0.57-1.56)	0.821
					CT	1.01 (0.70-1.44)	0.968
					TT	2.29 (0.76-6.89)	0.139
IRS1	rs1801278G>A	**5** (32, 34, 35, 41, 44)	5,371	6,255	AA	0.75 (0.17-3.33)	0.712
					GA	0.74 (0.58-0.94)	**0.016**
					GG	1.06 (0.82-1.37)	0.644
IRS2	rs1805097G>A	**5** (32, 34, 35, 41, 45)	5,220	6,014	AA	0.94 (0.84-1.06)	0.358
					GA	0.83 (0.71-0.96)	**0.013**
					GG	0.96 (0.84-1.10)	0.627

N, number; SNP, single nucleotide polymorphism, OR, odds ratio; CI, confidence interval; P, P-value.The bold values show P-value lower than 0.05 that is statistically significant.

**Figure 2:**
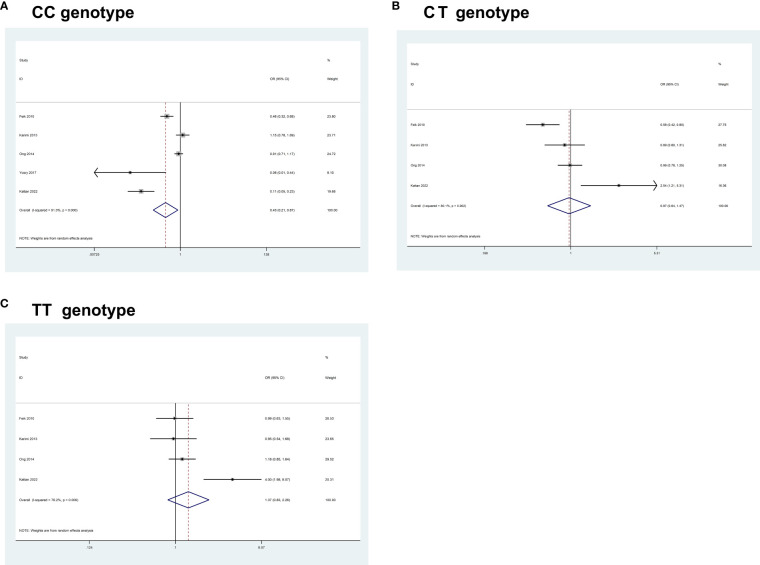
Forest plots of the association between *IGF1* rs6214C>T polymorphism and the risk of colorectal cancer. **(A)** CC genotype; **(B)** CT genotype; **(C)** TT genotype.

Overall, pooled results from 5 studies (comprising 1,035 cases and 2,726 controls) for *IGF1* rs6214C>T, revealed a significant association between the polymorphism and an increased CRC risk in some of the comparisons studied (CC, OR = 0.43, 95% CI 0.21- 0.87, P = 0.019; CT, OR = 0.97, 95% CI 0.64–1.47, P = 0.879; TT, OR = 1.37, 95% CI 0.83– 2.26, P = 0.216) (**Figure 2**).

Heterogeneity between study designs was obtained in the radial plot for *IGF1* rs6214C>T (CC, I^2 =^ 91%, P=0.000; CT, I^2 =^ 80.1%, P=0.002; TT, I^2 =^ 76.2%, P=0.006) (The results are not shown here). The pooled results based on 7 included studies for -969(CA) repeat (comprising 7,024 cases and 8,519 controls) and 6 eligible studies for *IGF1* rs35767C>T (comprising 3,434 cases and 5,698 controls) indicated that no significant association between these polymorphisms and CRC risk was found in any of the comparisons studied (**Table 6**).

**Figure 3:**
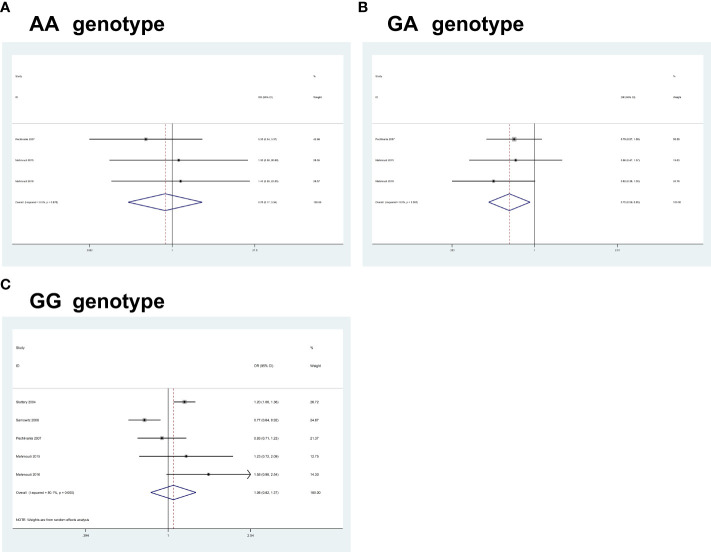
Forest plots of the association between *IRS1* rs1801278G>A polymorphism and the risk of colorectal cancer. **(A)** AA genotype; **(B)** GA genotype; **(C)** GG genotype.

For *IRS1* rs1801278G>A, pooled results based on 5 studies (comprising 5,371 cases and 6,255 controls) revealed a significant association between the polymorphism and an increased CRC risk in some of the comparisons studied (AA, OR = 0.75, 95% CI 0.17-3.33, P = 0.712; GA, OR = 0.74, 95% CI 0.58-0.94, P = 0.016; GG, OR = 1.06, 95% CI 0.82-1.37, P = 0.644) (**Figure 3**).

Heterogeneity between study designs was obtained in the radial plot for *IRS1* rs1801278G>A (AA, I^2 =^ 0.0%, P=0.676; GA, I^2 =^ 0.0%, P=0.691; GG, I^2 =^ 80.1%, P=0.000) (The results are not shown here).

**Figure 4:**
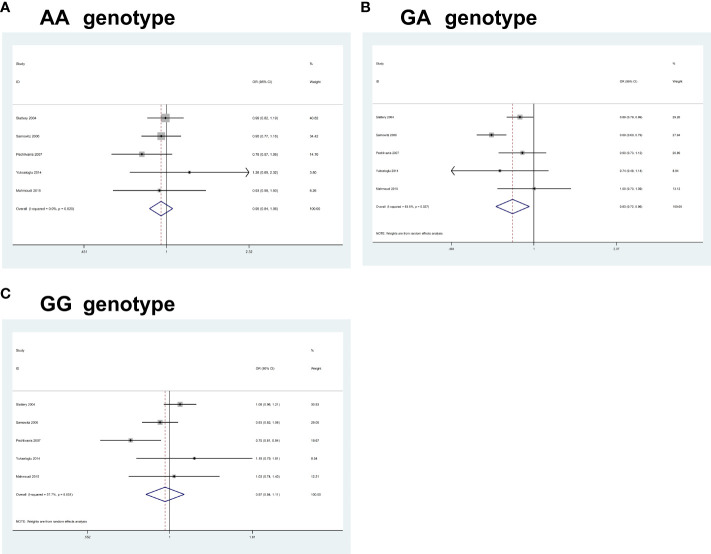
Forest plots of the association between *IRS2* rs1805097G>A polymorphism and the risk of colorectal cancer. **(A)** AA genotype; **(B)** GA genotype; **(C)** GG genotype.

For *IRS2* rs1805097G>A, pooled results from 5 studies (comprising 5,220 cases and 6,014 controls) revealed a significant association between the polymorphism and an increased CRC risk in some of the comparisons studied (AA, OR = 0.94, 95% CI 0.84-1.06, P = 0.358; GA, OR = 0.83, 95% CI 0.71-0.96, P = 0.013; GG, OR = 0.96, 95% CI 0.84-1.10, P = 0.627) (**Figure 4**). Heterogeneity between study designs was obtained in the radial plot for *IRS2* rs1805097G>A (AA, I^2 =^ 0.0%, P=0.620; GA, I^2 =^ 63.5%, P=0.027; GG, I^2 =^ 57.7%, P=0.051) (The results are not shown here).

#### Publication bias

The presence of publication bias was examined using Egger’s tests and visually verified using funnel plots. For rs6214C>T, no significant publication bias was detected according to the Egger’s test (CC, P=0.146; CT, P= 0.532; TT, P= 0.442). Similarly, no publication bias was detected for rs1801278G>A (AA, P= 0.029; GA, P= 0.729; GG, P= 0.980) and rs1805097G>A (AA, P= 0.902; GA, P= 0.733; GG, P= 0.866) (The results are not shown here). It is also noted that tests for funnel plot asymmetry should be used only when there are at least 10 studies included in the meta-analysis, because when there are fewer studies the power of the tests is too low to distinguish chance from real asymmetry. This was a limitation of the present study.

## Discussion

Colorectal cancer pathogenesis involves both genetic and environmental factors. As the effects of genetic mutations on CRC continued to be revealed, many authors have focused on the associations between SNPs and CRC susceptibility. There is a huge body of evidence on the implications of the IGF/insulin signaling pathway in the progression and development of cancers, which indicates an important prognostic factor for patients (53–55). This pathway activates the transformation-facilitating pathways of colorectal epithelial cells including proapoptotic and mitogenic signaling pathways (53). In particular, previous studies have brought strong evidence of the potential role of the IGF axis in the initiation and progression of cancer (56, 57). In other words, impairment in the regulation of the IGF axis considerably contributes to the malignancy phenotype by modifying cell behavior and boosting survival and invasion of cancer cells (58).

Over the decades, studies have been focused on identifying important genetic variations and SNPs of this pathway, which potentially influence the function of these genes. Despite being widely investigated, the relationship between the genes of the IGF axis and the risk of CRC is still poorly understood, and studies indicate conflicting findings. The contradicting results could attribute to the sample size of studies, ethnicity of participants, changes in the place of residence, lifestyle factors, and dietary patterns (59). For determining these heterogeneities, we designed a systematic study and meta-analysis according to the previous results, in order to achieve a more precise estimation of the correlation between the genes involved in the IGF pathway (*IGF1*, *IGF1R*, *IRS1*, and *IRS2*) and the risk of CRC for the first time.

One of the most important genes of this axis is *IGF1*, which may increase the risk of CRC through the regulation of cell proliferation, and apoptosis (23, 60). Epidemiological findings have indicated that elevation of serum concentration of IGF1 is related to increased risk of CRC because it can considerably increase the growth of cancer cells, suggesting evidence of the role of the IGF pathway in the risk and progression of carcinogenesis (7, 32, 61–63). Indeed, IGF1 is expressed locally in various tissues such as skeletal muscle and controls tissue growth *via* local paracrine and autocrine effects (64). Furthermore, it arouses cell proliferation and suppresses apoptosis (65). Therefore, it can be proposed that irregular expression of the *IGF1* gene leads to the progression of CRC (65, 66). There is evidence claiming that polymorphisms of the *IGF1* gene may be associated with the risk of CRC by affecting its serum level (40). Importantly, we showed that some polymorphisms especially *IGF1* rs35767C>T, and rs6214C>T CT and TT are associated with the serum IGF1 level (23, 40), so it makes sense to classify these polymorphisms as regulatory SNPs.

Our current findings showed that there was a significant correlation between polymorphism of *IGF1* rs6214C>T and the risk to develop CRC. However, there was a conflicting result regarding the relationship between *IGF* genotypes and the risk to develop CRC in previous studies. The significant association between rs6214C>T polymorphisms and risk of cancer development has been reported in various tumors including pancreatic, acute lymphocytic leukemia (ALL), esophageal, head and neck, and colorectal (38, 49, 67). Our result was in line with a previous study that revealed an association with cancer susceptibility in a population of Saudi Arabia, Egypt which clarified the correlation of rs6214C>T polymorphism with CRC susceptibility (38, 40). However, a meta-analysis study detect no significant relationship between rs6214C>T with overall cancer risk, but it had a significant correlation with breast and pancreatic cancer (68). Interestingly, we also observed that rs6214C>T polymorphism had a significant correlation with PFS.

Previous studies evaluated the association of *IGF1* rs35767C>T with the risk of different cancers which revealed contradicting results (23, 69, 70). Our meta-analysis proposed that *IGF1* rs35767C>T didn’t influence CRC risk. In contrast with our finding, a meta-analysis of 10 studies that included 9,415 CRC cases and 14,179 controls in Caucasians and Asian population showed that the *IGF1* rs35767C>T polymorphism was associated with decreased susceptibility to CRC in Caucasians, and has a protective effect against cancer (65). In a previous investigation, it was demonstrated that the *IGF1* CA-repeat polymorphism exhibited an association with an elevated susceptibility to develop CRC within the context of HNPCC (71). Nevertheless, Chen et al. conducted a meta-analysis and did not find a statistically significant correlation between *IGF1* (CA)n and the overall risk of cancer (72). Although, Qin et al. in a meta-analysis study declared that *IGF1* rs35767C>T polymorphism hadn’t a significant association with cancer risk (73). However, this result should be interpreted cautiously because studies on the *IGF1* rs35767C>T polymorphism exhibit high heterogeneity in terms of ethnicity and population. Additionally, this meta-analysis evaluated the association of *IGF1* rs35767C>T with several types of cancer in eight studies with 11,257 CRC patients and 16,213 healthy controls which only three of studies were about CRC (73).

*IGF1R* is predominately expressed in the digestive system and is involved in the proliferation of colon crypts (74). Considering the function of IGF1/IGF1R in cell differentiation, proliferation, and apoptosis, its activation is correlated with initiation, progression of cancer, and poor survival (53, 75). Thus, it can be supposed to be an attractive therapeutic target. Previous studies identified that various polymorphisms of the *IGF1R* gene could modify the susceptibility of cancer. Stanilov found that the *IGF1R* rs2229765G>A polymorphism was related to CRC progression; the allele A-carrying patients had higher levels of circulating IGF1 and were in higher stages of CRC compared to the GG genotype (48). Another study showed that serum concentration of IGF1 was higher in allele-A carrying *IGF1R* rs7166348 polymorphism and associated with a higher risk of colorectal neoplasm (76). To the contrary, in the current meta-analysis, we did not observe a significant correlation between *IGF1R* rs2229765G>A and the risk of developing CRC. On the other hand, the expression of IGF1R is regulated by P53 and P73. Thus, epigenetic modifications, deletion, and silencing of these proteins can impair the activation of *IGF1R* and lead to progression and metastasis in CRC (53).

IRSs are scaffold proteins, which mediate the IGF/insulin signaling pathway (77). They have critical roles in cell growth, proliferation, and cellular metabolism (77). Evidence has shown that these proteins play a significant role in the regulation of tumor development and progression of solid tumors (78). Several epidemiological studies have examined the relationship between IRSs polymorphisms and CRC risk (32, 79, 80). Based on our meta-analysis, a significant association was found between *IRS1* rs1801278G>A with the risk to develop CRC. Previous studies showed contradictory results for this polymorphism. In agreement with our findings, Su Yon Jung et al. demonstrated that the polymorphism of *IRS1* rs1801278G>A increases the risk of developing CRC by 30% in women who are inactive and consume exogenous estrogen (81). Similarly, Slattery reported a significant association between the *IRS1* rs1801278G>A (Gly972Arg) polymorphism and CRC risk in individuals using aspirin and NSAIDs. The G972R *IRS1* polymorphism has been linked to a 50% reduction in insulin sensitivity, suggesting that the GG genotype would reduce insulin resistance and the risk of CRC (81). However, a meta-analysis study conducted by Li et al. revealed that the *IRS1* rs1801278G>A polymorphism did not have a significant correlation with increased susceptibility of individuals to CRC (82) (**Table 4**).

Additionally, we found that *IRS2* rs1805097G>A significantly increased the risk of CRC. In line with our study, a meta-analysis found that *IRS2* rs1805097G>A polymorphism, leading to an aspartate replaced by glycine in the codon 1057 of its gene, changed the structure and function of the IRS2 protein and lowered the risk of overall CRC (78). Yin et al. conducted a meta-analysis comprising 6 case-control studies with a total of 4,333 cases and 5,333 controls, which demonstrated that the *IRS2* gene rs1805097G>A polymorphism plays a crucial role in the pathogenesis of CRC. This polymorphism was associated with a reduced risk of CRC, particularly colon cancer. Additionally, an ethnicity-based stratification analysis revealed that the rs1805097G>A polymorphism decreased the risk of CRC among Americans (83). In other words, the influence of this polymorphism on the manifestation of CRC depends upon the genetic background of individuals and the location of cancer within different populations. However, there were studies that reported no significant association between rs1805097G>A and CRC risk (21, 36, 41).

In our methodological systematic review, we managed to include all research related to our research questions and provided a quality assessment of each paper. To the best of our knowledge, this study represents a significant contribution to the current understanding of the relationship between IGF/insulin pathway polymorphisms and CRC risk. While previous investigations have explored the link between IGF1 gene polymorphisms and CRC risk, this study is among the few that have evaluated multiple major SNPs of this pathway in assessing CRC susceptibility. The findings of this study add to the existing literature on the subject matter and provide novel insights into the influence of the IGF/insulin pathway on individual susceptibility to CRC. Furthermore, we demonstrate links between different clinical outcomes such as PFS, OS, and drug response with multiple genetic variations in IGF axis pathway.

There are several limitations related to this study. First, our study was based on the studies published in indexed journals, which may increase bias related to time lag and publication bias. In the time-lag bias, literature with negative results compared to enthusiastic results is published over a long period of time (84). Concerning publication bias, studies with small sample sizes and negative results would not appear in the literature, while studies with small sample sizes and positive results are quickly published (85). Second, our search was restricted to English literature, and hence there was also an English language bias. Third, non-differential misclassification may exist because it is possible that the study’s control groups would develop cancer in the future. Finally, our data has been obtained according to the non-adjusted data. Thus, it seems that a more accurate adjusted analysis based on confounding factors such as age, tobacco, alcohol, and other environmental factors, can provide a precise estimate.

In addition, we were able to conduct a meta-analysis to quantify the contribution of genetic polymorphisms of *IGF1* (rs6214C>T), *IRS1* (rs1801278G>A), and *IRS2* (rs1805097G>A) to CRC risk. Of note, other genetic variations in *IGF1*, *IGFR1*, *IRS1*, and *IRS2* were not included in meta-analysis due to heterogeneity and small sample size.

## Conclusion and future perspective

In summary, our findings indicated that genotypes of CC in *IGF1* rs6214C>T, and GA in *IRS1* rs1801278G>A, and *IRS2* rs1805097G>A are associated with an increased risk of CRC which can serve as diagnostic biomarkers in CRC. Indeed, the identification of specific genetic variants associated with an increased risk of CRC could inform future research on prevention and treatment strategies for this disease. These findings may also facilitate the development of personalized medicine approaches, allowing for targeted interventions in at-risk populations. -

Thus, given the limitation points of this study and practical reasons, epidemiological studies with a larger sample scale evaluating various populations as well as incorporating more comprehensive and accurate assessments are required to validate the findings of this study. However, there is still limited information regarding early detection of CRC, and gene-environment and gene-gene interactions must also be considered in succeeding research to apply early detection of CRC.

## Data availability statement

The data analyzed in this study is subject to the following licenses/restrictions: <b>No additional data available.</b>. Requests to access these datasets should be directed to <b>n_fatemi_1363@yahoo.com</b>.

## Author contributions

MC and NF conceived and designed the study. MC, MA, AS and NF conducted systematic search, screened articles, and selected eligible articles. PM, MK, EN-M and MP extracted the result from included study and performed eligible evaluation. MC, NF, MA, HA and SS performed analysis and interpreted the findings. MC and NF wrote the first version of the manuscript. ST, AS, HA and EN-M revised the manuscript. All authors read and approved the final version of the manuscript.
