# Supplementary material for: A systematic review and meta-analysis for the association of the insulin-like growth factor1 pathway genetic polymorphisms with colorectal cancer susceptibility
Source: Front Oncol. 2023 May 22;13:1168942. doi: 10.3389/fonc.2023.1168942 (PMC10240407; doi:10.3389/fonc.2023.1168942)
Supplement: Supplementary file 1 [file Table_1.docx]

Supplementary Table 1. Results from the Critical Appraisal Skills Programme quality assessment checklist for case control studies.

| Study | Did the study address a clearly focused issue? | Did the authors use an appropriate method to answer their question? | Were the cases recruited in an acceptable way? | Were the controls selected in an acceptable way? | Was the exposure accurately measured to minimize bias? | Have the authors taken account of the potential confounding factors in the design and/or in their analysis? | Do you believe the results? | Can the results be applied to the local population? | Do the results of this study fit with other available evidence? | How valuable is the research? |
| --- | --- | --- | --- | --- | --- | --- | --- | --- | --- | --- |
| Wong, H L; et al.2005 (29) | Yes | Yes | Yes | Yes | Yes | Yes | Yes | Yes | Can’t Tell | Valuable |
| Martha L Slattery et al, 2004 (31) | Yes | Yes | Yes | Yes | Yes | Yes | Yes | Yes | Yes | Valuable |
| [Libby M Morimoto et al, 2005](https://pubmed.ncbi.nlm.nih.gov/?term=Morimoto+LM&cauthor_id=15894673) (32) | Yes | Yes | Yes | Yes | Yes | Yes | Yes | Yes | Yes | Valuable |
| [Wade S Samowitz et al, 2006](https://pubmed.ncbi.nlm.nih.gov/?term=Samowitz+WS&cauthor_id=16448675) (33) | Yes | Yes | Yes | Yes | Yes | Yes | Yes | Yes | Yes | Valuable |
| Sonali Pechlivanis et al, 2007 (34) | Yes | Yes | Yes | Yes | Yes | Yes | Yes | Yes | Yes | Valuable |
| Sonali Pechlivanis et al, 2007 (35) | Yes | Yes | Yes | Yes | Yes | Yes | Yes | Yes | Yes | Valuable |
| Ayman Yosry et al, 2017 (37) | Yes | Yes | Yes | Yes | Yes | Yes | Yes | Yes | Yes | Valuable |
| Elisabeth Feik et al, 2010 (38) | Yes | Yes | Yes | Yes | Yes | Yes | Yes | Yes | Yes | Valuable |
| Shahad W. Kattan et al, 2022 (39) | Yes | Yes | Yes | Yes | Yes | Yes | Yes | Yes | Yes | Valuable |
| Touraj Mahmoudi et al, 2015 (40) | Yes | Yes | Yes | Yes | Yes | Yes | Yes | Yes | Yes | Valuable |
| Khatoon Karimi et al, 2013 (42) | Yes | Yes | Yes | Yes | Yes | Yes | Yes | Yes | Yes | Valuable |
| Touraj Mahmoudi et al, 2016 (43) | Yes | Yes | Yes | Yes | Yes | Yes | Yes | Yes | Yes | Valuable |
| Emel Hulya Yukseloglu et al, 2014 (44) | Yes | Yes | Yes | Yes | Yes | Yes | Yes | Yes | Yes | Valuable |
| Nicholas J Ollberding et al, 2012 (45) | Yes | Yes | Yes | Yes | Yes | Yes | Yes | Yes | Yes | Valuable |
| H-L Wong et al, 2008 (46) | Yes | Yes | Yes | Yes | Yes | Yes | Yes | Yes | Yes | Valuable |
| Noyko S Stanilov, et al, 2014 (47) | Yes | Yes | Yes | Yes | Yes | Yes | Yes | Yes | Yes | Valuable |
| Jennie Ong et al, 2014 (48) | Yes | Yes | Yes | Yes | Yes | Yes | Yes | Yes | Yes | Valuable |
| Temitope O Keku et al, 2012 (19) | Yes | Yes | Yes | Yes | Yes | Yes | Yes | Yes | Yes | Valuable |
| Xianyang Li et al, 2022 (49) | Yes | Yes | Yes | Yes | Yes | Yes | Yes | Yes | Yes | Valuable |
| Yang Li et al, 2018 (21) | Yes | Yes | Yes | Yes | Yes | Yes | Yes | Yes | Yes | Valuable |
| X L Chao et al, 2019 (50) | Yes | Yes | Yes | Yes | Yes | Yes | Yes | Yes | Yes | Valuable |

Supplementary Table 2. Results from the Critical Appraisal Skills Programme quality assessment checklist for cohort studies.

| Study | Did the study address a clearly focused issue? | Was the cohort recruited in  an acceptable way? | Was the exposure accurately  measured to minimise bias? | Was the outcome accurately  measured to minimise bias? | Have the authors identified  all important confounding  factors? | Have they taken account of  the confounding factors in the  design and/or analysis? | Was the follow up of  subjects complete enough? | Was the follow up of  subjects long enough? | Do you believe the results? | Can the results be applied to  the local population? | Do the results of this study fit  with other available  evidence? | What are the implications of  this study for practice? | How valuable is the research? |
| --- | --- | --- | --- | --- | --- | --- | --- | --- | --- | --- | --- | --- | --- |
| Jen-Kou Lin, et al. 2010 (30) | Yes | Yes | Yes | Yes | Yes | Yes | Yes | Can’t Tell | Yes | Yes | Yes | Yes | Valuable |
| Armin Gerger et al, 2011 (36) | Yes | Yes | Yes | Yes | Yes | Yes | Yes | Yes | Yes | Yes | Yes | Yes | Valuable |
| Yoon Young Cho et al, 2011 (41) | Yes | Yes | Yes | Yes | Yes | Yes | Yes | No | Yes | Yes | Yes | Yes | Valuable |
| Colinda C J M Simons et al, 2015 (20) | Yes | Yes | Yes | Yes | Yes | Can’t Tell | Yes | Yes | Can’t Tell | Can’t Tell | No | Yes | Valuable |
| Thomas Winder et al, 2010 (51) | Yes | Yes | Yes | Yes | Yes | Can’t Tell | Yes | Yes | Can’t Tell | Can’t Tell | No | Yes | Valuable |
