# Supplementary material for: A systematic review and meta-analysis for the association of the insulin-like growth factor1 pathway genetic polymorphisms with colorectal cancer susceptibility
Source: Front Oncol. 2023 May 22;13:1168942. doi: 10.3389/fonc.2023.1168942 (PMC10240407; doi:10.3389/fonc.2023.1168942)
Supplement: Supplementary file 2 [file Table_2.docx]

PubMed: 89

(IGF1R OR “Insulin-Like Growth Factor 1 Receptor” OR “IGF-1” OR “IGF-I” OR “Insulin Like Growth Factor I” OR IGF1 OR “Insulin-Like Growth Factor 1” OR “IGF-1 Receptor” OR “IGF 1 Receptor” OR “IGF-I Receptor” OR “IGF I Receptor” OR “Insulin-Like-Growth Factor I Receptor” OR “Insulin Like Growth Factor I Receptor” OR “IGF Type 1 Receptor” OR (Receptor AND IGF-I) OR (Receptor AND IGF I) OR (Receptor AND IGF-1) OR (Receptor AND “Insulin-Like Growth Factor Type 1”) OR (Receptor AND “Insulin-Like Growth Factor I”) OR “IRS Signaling Adaptor Protein” OR “Insulin Receptor Substrate-1” OR “Insulin Receptor Substrate 1” OR “Insulin Receptor Substrate” OR IRS) AND (Colorectal OR “Colon Cancer” OR “colonic carcinoma" OR “Colon Adenocarcinoma” OR “Rectal Cancer” OR CRC OR “colorectal carcinoma” OR "colon carcinoma”) AND ("genetic variation single nucleotide polymorphism" OR "single nucleotide polymorphism" OR snp OR polymorphism) AND (1998-2022.06.30)

Wos:151

(IGF1R OR “Insulin-Like Growth Factor 1 Receptor” OR “IGF-1” OR “IGF-I” OR “Insulin Like Growth Factor I” OR IGF1 OR “Insulin-Like Growth Factor 1” OR “IGF-1 Receptor” OR “IGF 1 Receptor” OR “IGF-I Receptor” OR “IGF I Receptor” OR “Insulin-Like-Growth Factor I Receptor” OR “Insulin Like Growth Factor I Receptor” OR “IGF Type 1 Receptor” OR (Receptor AND IGF-I) OR (Receptor AND IGFI) OR (Receptor AND IGF-1) OR (Receptor AND “Insulin-Like Growth Factor Type 1”) OR (Receptor AND “Insulin-Like Growth Factor I”) OR “IRS Signaling Adaptor Protein” OR “Insulin Receptor Substrate-1” OR “Insulin Receptor Substrate 1” OR “Insulin Receptor Substrate” OR IRS) AND (“Colorectal” OR “Colon Cancer” OR “colonic carcinoma" OR “Colonic Cancer” OR “Colon Adenocarcinoma” OR “Rectal Cancer” OR CRC OR “colorectal carcinoma” OR "colon carcinoma”) AND ("genetic variation single nucleotide polymorphism" OR "single nucleotide polymorphism" OR snp OR polymorphism)

SCOPUS: 2380

(TITLE-ABS-KEY(IGF1R) OR TITLE-ABS-KEY(“Insulin-Like Growth Factor 1 Receptor”) OR TITLE-ABS-KEY(“IGF-1”) OR “IGF-I” OR “Insulin Like Growth Factor I” OR IGF1 OR “Insulin-Like Growth Factor 1” OR “IGF-1 Receptor” OR “IGF 1 Receptor” OR “IGF-I Receptor” OR “IGF I Receptor” OR “Insulin-Like-Growth Factor I Receptor” OR TITLE-ABS-KEY(“Insulin Like Growth Factor I Receptor”) OR “IGF Type 1 Receptor” OR (Receptor AND IGF-I) OR (Receptor AND IGFI) OR (Receptor AND IGF-1) OR (Receptor AND “Insulin-Like Growth Factor Type 1”) OR (Receptor AND “Insulin-Like Growth Factor I”) OR “IRS Signaling Adaptor Protein” OR “Insulin Receptor Substrate-1” OR TITLE-ABS-KEY(“Insulin Receptor Substrate 1”) OR TITLE-ABS-KEY(“Insulin Receptor Substrate”) OR IRS) AND (TITLE-ABS-KEY(“Colorectal”) OR TITLE-ABS-KEY(“Colon Cancer”) OR TITLE-ABS-KEY(“colonic carcinoma") OR “Colonic Cancer” OR “Colon Adenocarcinoma” OR “Rectal Cancer” OR CRC OR TITLE-ABS-KEY(“colorectal carcinoma”) OR TITLE-ABS-KEY("colon carcinoma”)) AND ("genetic variation single nucleotide polymorphism" OR "single nucleotide polymorphism" OR snp OR polymorphism)
